# Improved Functionality and Emulsifying Stability of Chickpea Protein Microgel–Mung Bean Hull Fiber Conjugates via Combined Physicochemical Pretreatment

**DOI:** 10.3390/foods15193526

**Published:** 2026-10-02

**Authors:** Xiangxiang He, Xinyi Song, Liping Ma, Jinle Xiang, Min Zhang, Xinshuai Zhang, Yizhuo Han, Yunpeng Shao, Runqiang Yang, Lei Luo, Bo Zhang

**Affiliations:** 1 College of Food and Bioengineering, Henan University of Science and Technology, Luoyang 471000, China; xianghe2009@126.com (X.H.); songxiyi2001@163.com (X.S.); maliping226@163.com (L.M.); xjl5013@haust.edu.cn (J.X.); 9901840@haust.edu.cn (M.Z.); hnzhangxinshuai@163.com (X.Z.); han0701042026@163.com (Y.H.); 18322012469@163.com (Y.S.); 2 College of Food Science & Technology, Nanjing Agricultural University, Nanjing 210095, China; yangrq@njau.edu.cn; 3 College of Basic Medicine, Zunyi Medical University, Zunyi 563000, China

**Keywords:** chickpea protein, microgel, high-pressure homogenization, ultrasound, Maillard reaction, functionality, emulsifying stability

## Abstract

Native chickpea protein isolate (CPI) exhibits unsatisfactory solubility and dispersibility, restricting its interfacial performance and emulsion-stabilizing capacity. Herein, CPI gels were subjected to combined high-pressure homogenization and ultrasonication pretreatment to fabricate CPI microgel particles (CPIMPs), followed by a Maillard conjugation with mung bean hull soluble dietary fiber (MSDF). Then the effects of glycosylated CPIMP–MSDF conjugates on structural properties, functional properties, and emulsion stability were evaluated. The results demonstrated that the grafting degree of the CPIMP–MSDF conjugate reached a peak of 35.54% at a 4:1 protein-to-polysaccharide mass ratio, indicating efficient grafting. FTIR, intrinsic fluorescence spectroscopy, and free sulfhydryl content analysis confirmed the structural changes. Glycosylation was found to significantly enhance the antioxidant activity, foaming properties, and emulsifying capacity of conjugates. The emulsifying activity index (EAI) and emulsion stability index (ESI) of the CPIMP–MSDF conjugates were 2.52- and 2.91- fold higher than those of CPI, respectively. The Pickering emulsion stabilized by the CPIMP–MSDF conjugate exhibited the smallest droplet size (4.72 μm) and the lowest zeta potential (−30.71 mV). Furthermore, glycosylation significantly enhanced the storage stability and thermal stability of the Pickering emulsions. This combined physical pretreatment and Maillard conjugation provides a feasible strategy to upgrade the functional attributes of legume protein.

## 1. Introduction

Plant-based proteins have emerged as ideal substitutes for animal-derived proteins, owing to their sustainability and multifunctionality, and have assumed an increasingly prominent role in human dietary patterns [1]. Plant proteins originate from a wide variety of sources, such as cereals, oilseeds, nuts and legumes. Among these, the chickpea, the third most widely grown legume globally, has a protein content of 14.9–24.6%. Chickpea protein isolate (CPI), in particular, possesses a well-balanced amino acid profile and favorable bioavailability, rendering it a highly promising plant-based protein ingredient for functional food development and utilization [2]. As an amphiphilic biopolymer, CPI is intrinsically capable of adsorbing at oil-water interfaces and functioning as an emulsifier. However, CPI is predominantly composed of globulins, albumins, prolamins, and glutelins, while the globulin fraction has a content ranging from 53% to 60% [3]. Its molecular structure adopts a highly compact and rigid globular conformation, which impairs its unfolding capacity and solubility in aqueous media. Moreover, as a native globular protein, CPI has most of its hydrophobic side chains buried within its molecular core, which restricts its rapid adsorption and conformational rearrangement at the oil-water interface [4]. This insufficient interfacial activity hinders CPI from stabilizing emulsions effectively and compromises its environmental tolerance.

The modification of proteins to improve their poor solubility, imbalanced amphiphilicity, and environmental stability is critical for the expansion of their application in food-related fields. Various modification techniques, including physical approaches such as cold plasma treatment [5], high-pressure homogenization [6], and ultrasonication [7], chemical approaches including acylation [8], deamidation [9] and glycosylation [10], as well as biological approaches such as enzymatic hydrolysis [11], serve to improve the physicochemical and functional properties of plant proteins. Among these methods, glycosylation through the Maillard reaction stands out, owing to its covalent conjugation capability and environmentally friendly nature [12]. Glycosylation modification induces significant structural changes in proteins, which confer multiple benefits in emulsion stabilization. For instance, rice protein glycosylated with dextran exhibited substantially improved solubility, emulsifying capacity, and foaming properties [13]. Conjugation with hydrophilic polysaccharide chains elevates the steric repulsion of proteins molecules, thereby effectively inhibiting droplet coalescence and reinforcing emulsion resilience [14].

The compact molecular conformation of native CPI limits the accessibility of reactive amino groups, thereby restricting the grafting efficiency. Although various pretreatments have been employed to modify protein structures for improved glycosylation, including ultrasound-assisted glycation of chickpea protein isolate for Pickering emulsion characterization [15], the combined use of sequential high-pressure homogenization and ultrasonication to prepare CPI microgels (CPIMPs) for enhanced grafting efficiency has been rarely reported. Studies have demonstrated that ultrasonication can induce protein unfolding through mechanical, thermal, and cavitation effects, thereby exposing more reactive amino groups and promoting glycosylation [15]. High-pressure homogenization treatment can disrupt protein aggregates and alter their molecular conformation, thereby enhancing protein solubility and reactivity [6]. Additionally, soft microgel particles fabricated by physical shearing can effectively extend polypeptide chains and have been employed as a pretreatment prior to glycosylation [16]. Based on these considerations, it is proposed that combined sequential homogenization and ultrasonication pretreatment for the preparation of CPIMPs can effectively unfold the polypeptide chains of CPI and improve its grafting degree.

It has been reported that monosaccharides or polysaccharides are commonly selected as sugar donors in the Maillard reaction. Typical monosaccharides include glucose [17], ribose [18], and galactose [19], while polysaccharides commonly used include pectin [20], dextran [21], sodium alginate [22], and others. Compared with small-molecule monosaccharides, polysaccharides possess larger molecular dimensions and more complex branched structures. These structural characteristics confer stronger steric hindrance effects in the protein glycosylation products and thus make them more suitable for such applications [23]. In this study, soluble dietary fiber from mung bean hull (MSDF) was selected as the sugar donor, offering multiple advantages. MSDF, a complex natural polysaccharide containing arabinose, galactose, glucose, fructose, and others, can provide the reducing carbonyl groups required for the Maillard reaction [24]. Moreover, MSDF contains certain amounts of bioactive compounds, such as flavonoids and polyphenols, which may boost the antioxidant capacity of the resulting glycosylated conjugates [25].

Our explicit hypothesis was that combined high-pressure homogenization and ultrasonication pretreatment could disrupt the compact native conformation of CPI, expose more reactive amino groups of polypeptide chains, and consequently promote Maillard-mediated covalent grafting between CPIMPs and MSDF. The obtained CPIMP–MSDF conjugates were expected to exhibit improved functional performance and Pickering emulsion stability. In this study, CPI was processed into a gel, which was then treated with the combined application of high-pressure homogenization and ultrasonication to obtain CPIMPs, thereby promoting the glycosylation reaction of CPIMPs with MSDF. The structural alterations of CPI induced by glycosylation were systematically evaluated by determining the degree of graft, FTIR spectroscopy, intrinsic fluorescence spectroscopy, as well as free sulfhydryl content. Meanwhile, the foamability, antioxidant activity, and emulsifying properties of the resulting CPIMP–MSDF conjugate were evaluated. Furthermore, the stability of the CPIMP–MSDF conjugate-stabilized oil-in-water (O/W) Pickering emulsion was systematically characterized. Collectively, this study provides new perspectives for improving the functionality and emulsifying capacity of CPI, which is expected to facilitate the development of CPI as a natural emulsifier and broaden its development prospects in food and emulsion-related fields.

## 2. Materials and Methods

### 2.1. Materials

Chickpea protein isolate (CPI) was obtained from Shaanxi Yutai Biotechnology Co., Ltd. (Xi’an, China). Its measured proximate composition was as follows: protein content (dry basis): 82.41 ± 0.63%, moisture: 6.28 ± 0.21%, lipid: 1.74 ± 0.12%, and ash: 2.36 ± 0.09%. Mung bean hull was sourced from Luoyang New Countryside Vegetables Foods Co., Ltd. (Luoyang, China). Transglutaminase (TGase, 100 U/g) was purchased from Zhongxing Biotechnology Co., Ltd. (Zhengzhou, China), while medium chain triglyceride (MCT), cellulase (400 U/g), papain (800 U/mg) and amyloglucosidase (100 U/mg) were obtained from Shanghai Yuanye Bio-Technology Co., Ltd. (Shanghai, China). All reagents are analytical grade.

### 2.2. Preparation of Chickpea Protein Isolate Microgel Particles (CPIMPs)

CPIMPs were prepared following a method reported in earlier studies, with some adjustments [26]. Firstly, CPI powder was dispersed into deionized water to form a CPI dispersion (12 wt%). The dispersion was continuously agitated at 400 rpm for 2 h and subsequently kept at 4 °C overnight to ensure that CPI protein molecules were fully hydrated. The CPI dispersion was magnetically stirred in a water bath at 85 °C for 30 min to unfold the compact conformation of CPI, followed by natural cooling down to 45 °C. Transglutaminase (TGase) was added to the cooled CPI dispersion and mixed uniformly (20 U/g CPI). The mixture was incubated at constant 45 °C for 4 h without stirring to form the CPI gel. Subsequently, the obtained CPI gel was mixed with two volumes (*v*/*w*) of pre-cooled deionized water, then subjected to high-speed shearing at 12,000 rpm for 3 min using a disperser (AD500S-H, Shanghai Angni Instruments Co., Ltd., Shanghai, China). The pre-sheared sample was further homogenized for 5 cycles at 45 MPa using a high-pressure homogenizer (AH-BASIC II, ATS Engineering Inc., Cambridge, ON, Canada). Afterwards, the sample was treated with a probe ultrasonic processor (JY92-IIN, NingBo Scientz Biotechnology Co., Ltd., Ningbo, China) at 200 W for 40 min (5 s on/3 s off pulse mode). During ultrasonication, the sample container was immersed in an ice-water bath to avoid excessive thermal accumulation. Finally, a 4 wt% CPIMP dispersion was obtained.

### 2.3. Preparation of Mung Bean Hull Soluble Dietary Fiber (MSDF)

MSDF was prepared from mung bean hulls using the enzymatic extraction procedure [27], with minor adjustments. The washed mung bean hulls were dried in a drying oven, then ground into powder (100 mesh) using a high-speed crusher (FW-400A, Beijing Zhongxingweiye Instrument Co., Ltd., Beijing, China). A 10 g sample of mung bean hull powder was suspended in deionized water (1:15, *w*/*w*). The dispersion was subjected to sequential enzymatic hydrolysis using 80 mg of cellulase at 50 °C for 4 h (pH 5.0), 50 mg of papain at 60 °C for 30 min (pH 5.0), and finally 100 mg of amyloglucosidase at 60 °C for 30 min (pH 4.5). The enzymes were then inactivated by heating the mixture in a boiling water bath for 10 min and proteins were removed using the Sevag reagent. The mixture was centrifuged for 20 min at 4000× *g* rpm. The supernatant containing MSDF was then collected and subjected to ethanol precipitation. Finally, the precipitate was freeze-dried and then collected for storage.

### 2.4. Preparation of CPIMP–MSDF Conjugate

CPIMP–MSDF conjugates were obtained using the wet-heat Maillard reaction method [28]. 4% CPIMP solution were mixed with different concentrations of MSDF solutions in equal volumes to obtain CPIMP–MSDF mixtures at various mass ratios of 16:1, 8:1, 4:1, 2:1 and 1:1. The pH values of the CPIMP–MSDF mixtures with different concentrations were adjusted to 10 using NaOH solution. Afterwards, the resulting mixtures were heated at 95 °C for 6 h to produce CPIMP–MSDF conjugates.

### 2.5. Degree of Graft (DG) Measurements and Structural Characterizations

#### 2.5.1. Determination of DG and Browning Intensity

The DG of CPIMP–MSDF conjugates was measured via the o-phenyl-enediamine (OPA) method [29]. The OPA reagent was freshly prepared by dissolving 80 mg of o-phthalaldehyde in 2 mL methanol, followed by mixing with 50 mL of 0.1 M sodium tetraborate buffer (pH 9.5), 5 mL of SDS solution (20%, *w*/*v*), and 20 μL of β-mercaptoethanol. A 200 μL aliquot of sample solution (1 mg/mL) was added to 4 mL of OPA reagent, followed by incubation at 35 °C for 2 min. Subsequently, the absorbance was recorded at 340 nm with a UV-Vis spectrophotometer (T6 New Century, Beijing Purkinje General Instrument Co., Ltd., Beijing, China), where the blank consisted of distilled water and OPA reagent. The DG was determined using the following formula:

(1) $$\mathrm{DG}\left(\%\right)=\frac{A_{0}-A_{t}}{A_{0}}\times 100$$

where A_0_ represents the absorbance of the solution before glycosylation, and Aₜ represents that after glycosylation.

For browning intensity evaluation of the Maillard reaction, the CPIMP–MSDF conjugate dispersions were adjusted to 0.5 mg/mL, and their absorbance was recorded at 420, 304, and 290 nm.

#### 2.5.2. Measurement of Zeta Potential and Particle Size

The zeta potential of the sample dispersions was measured using a nanoparticle size and zeta potential analyzer (BeNano 90 Zeta, Dandong Bettersize Instruments Ltd., Dandong, China), and the particle size distribution was measured using a laser diffraction particle size analyzer (Bettersize2600, Dandong Bettersize Instruments Ltd., Dandong, China). All measurements were carried out at 25 °C.

#### 2.5.3. Measurement of Fourier Transform Infrared (FTIR) Spectra

FTIR spectra of the samples were recorded with a Fourier transform infrared spectrometer (Vector33, Bruker Corporation, Karlsruhe, Germany) over the wavenumber range of 400–4000 cm^−1^ [30]. The sample solutions were freeze-dried to obtain solid powders. Subsequently, the dried powders were uniformly mixed with KBr at a mass ratio of 1:100 and compressed into pellets for FTIR measurement. The protein’s secondary structure was fitted and evaluated via OMNIC 8.2 and PeakFit v4.12 software.

#### 2.5.4. Measurement of Intrinsic Fluorescence Spectra

The intrinsic fluorescence spectra of the samples (diluted to 5 mg/mL) were obtained by a fluorescence spectrophotometer (Cary Eclipse, Agilent Technologies, Inc., Santa Clara, CA, USA). Fluorescence emission spectra were recorded over a range of 290–500 nm with excitation at 280 nm and a slit width of 5 nm.

#### 2.5.5. Measurement of Free Sulfhydryl Content

Following Ellman & Lysko’s procedure, the free sulfhydryl content was measured. Each 1 mL sample solution (2 mg/mL) was mixed with 1 mL Tris-glycine buffer (pH 8.0) composed of 0.086 mol/L Tris, 0.09 mol/L glycine, and 0.04 mol/L disodium EDTA. Then, 20 μL of DNTB solution (4 mg/mL) was added to the mixed solution, followed by incubation at 4 °C for 1 h. The resulting mixture was centrifuged at 3500× *g* rpm for 30 min, and the absorbance of the supernatant was recorded at 412 nm using a UV-Vis spectrophotometer (T6 New Century, Beijing Purkinje General Instrument Co., Ltd., Beijing, China). The sulfhydryl concentration was quantified according to the formula below:

(2) $$\mathrm{Free sulfhydryl content}=\frac{73.53\times A\times \mathrm{DF}}{C}$$

where the free sulfhydryl content is expressed in units of μmol/g, and A, DF, and C are the absorbance, dilution factor, and mass concentration (mg/mL) of the sample, respectively.

### 2.6. Functional Characterizations

#### 2.6.1. Measurement of DPPH• Radical Scavenging Activity

The DPPH• radical scavenging activity of the samples was evaluated according to the method of Dong et al. [31]. The sample was diluted to 2 mg/mL, and a 1 mL aliquot was added to 5 mL of DPPH• solution (0.1 mM). The mixture was allowed to react in the dark for 30 min, and the absorbance was read at 517 nm with a UV-Vis spectrophotometer. The DPPH• radical scavenging activity of samples was calculated according to the following formula:

(3) $$\mathrm{DPPH}•\mathrm{radical scavenging activity}\left(\%\right)=\left(1-\frac{A_{S}-A_{C}}{A_{0}}\right)\times 100\%$$

A_S_ represents the absorbance of the reacted sample, A_C_ corresponds to that of the sample without DPPH• solution, and A_0_ refers to that of the blank (composed of anhydrous ethanol and DPPH• solution).

#### 2.6.2. Measurement of Emulsifying Properties

The emulsifying activity index (EAI) and emulsifying stability index (ESI) of the samples were evaluated according to a previously reported method [32]. Briefly, 16 mL of the sample dispersion (10 mg/mL) was mixed with 4 mL of MCT, and the mixture was emulsified at 12,000 rpm for 3 min using an AD500S-H dispenser (Shanghai Angni Instruments Co., Ltd., Shanghai, China). After emulsification for 0 and 20 min, 20 µL of the bottom emulsion layer was diluted in 10 mL of 0.1% SDS solution, and the absorbance was immediately measured at 500 nm. The EAI and ESI were determined using the formulas below:

(4) $$\mathrm{EAI}\left(\frac{m^{2}}{g}\right)=\frac{2\times 2.303\times A_{0}\times \mathrm{DF}}{\theta \mathrm{Lc}\times 10^{4}}$$

(5) $$\mathrm{ESI}\left(\min\right)=\frac{A_{0}\times 20}{A_{0}-A_{20}}$$

where A_0_ and A_20_ are the absorbance values of the diluted emulsion measured at 0 min and 20 min after emulsification, respectively. DF is the dilution factor with SDS solution; c is the protein concentration (g/mL); θ is the oil volume fraction (*v*/*v*); and L is the optical path length of the colorimetric cell (1 cm). ESI represents the predicted time required for the emulsion to lose half of its turbidity.

#### 2.6.3. Measurement of Foaming Properties

The foam ability and foam stability of the sample were evaluated following a previously reported method, with a slight modification [33]. A certain volume of sample solution was transferred into a graduated cylinder, and the initial liquid volume was recorded as V_0_ (mL). After high-speed stirring for 1 min (5000 rpm), the total volume was immediately recorded as V_1_ (mL). Subsequently, the total volume was measured again after standing for 30 min as V_30_ (mL). The foamability and foam stability were calculated using the equations below:

(6) $$\mathrm{Foamability}(\%)\;=\;\frac{V_{1}-V_{0}}{V_{0}}\;\times \;100$$

(7) $$\mathrm{Foam}\;\mathrm{stability}(\%)=\frac{{V_{30}-V}_{0}}{V_{0}}\;\times \;100$$

### 2.7. Characterization Emulsion Stabilized by CPIMP–MSDF Conjugate

#### 2.7.1. Preparation of Emulsions

Sample solutions (2 wt%) were prepared according to the method described above and mixed with MCT at a water-to-oil ratio of 8:2 (*v*/*v*). The Pickering emulsions were then prepared using an AD500S-H disperser (Shanghai Angni Instruments Co., Ltd., Shanghai, China) under 12,000 rpm for 4 min.

#### 2.7.2. Microstructure Observation

Confocal laser scanning microscopy (CLSM): The morphology and structure of the emulsions were visualized using a confocal laser scanning microscope (FV3000, Olympus Co., Tokyo, Japan). For staining, 20 μL of Nile Red solution (excitation wavelength λ = 488 nm) and 20 μL of Nile Blue solution (excitation wavelength λ = 633 nm) were added to 1 mL of the emulsion sample and thoroughly mixed to label the oil and protein phase. The stained sample was observed for its microstructure under 400× magnification.

Optical microscopy: To capture optical microscopic images of droplet morphology, emulsion samples were placed onto glass slides and then viewed under a fluorescence microscope (EVOS™ M7000, Thermo Fisher Scientific, Waltham, MA, USA) with a 40× objective lens.

#### 2.7.3. Particle Size and Zeta Potential of Emulsions

The average droplet size and size distribution of the different emulsions were determined using a Bettersize2600 particle size analyser(Dandong Bettersize Instruments Ltd., Dandong, China). The zeta potential of the emulsions was measured using a BeNano 90 Zeta analyzer (Dandong Bettersize Instruments Ltd., Dandong, China). Triplicate measurements were averaged for each sample.

#### 2.7.4. Rheological Measurements 

A Discovery Hybrid Rheometer-2 (DHR-2, TA Instruments, New Castle, DE, USA) with a φ40 mm parallel plate geometry was used to determine the storage modulus (G’), loss modulus (G”), and apparent viscosity (η) of the emulsions. All tests were performed at 25 °C with a gap of 1000 μm. Frequency sweep tests were performed at a fixed strain of 1% from 1 to 100 rad/s to determine G’ and G”. In addition, the apparent viscosity was determined under steady-state shear at rates from 0.1 to 100 s^−1^.

#### 2.7.5. Stability of Emulsions

The storage stability of the emulsions was assessed by measuring droplet size and zeta potential over a 14-day storage period at 4 °C, with measurements taken on days 0, 3, 5, 7, and 14. Thermal stability was evaluated respectively by incubating freshly prepared emulsions at 25, 50, 75, and 100 °C for 3 h, followed by the same measurements.

### 2.8. Statistical Analysis

All measurements were carried out in triplicate, and the resulting data are given as means ± standard deviations (SD). Statistical evaluation was performed using SPSS 18.0 (SPSS Inc., Chicago, IL, USA). For data with a single independent variable, one-way ANOVA followed by Duncan’s multiple range test was applied at a significance level of *p* < 0.05. For storage stability and thermal stability datasets containing two independent variables, two-way ANOVA was performed to evaluate the main effects and factor interaction. At each fixed time or temperature, one-way ANOVA followed by Duncan’s multiple range test was used for pairwise comparisons (*p* < 0.05).

## 3. Results and Discussion

### 3.1. Structural Characterizations of CPIMP–MSDF Conjugate

#### 3.1.1. DG and Browning Intensity

**Figure 1:**
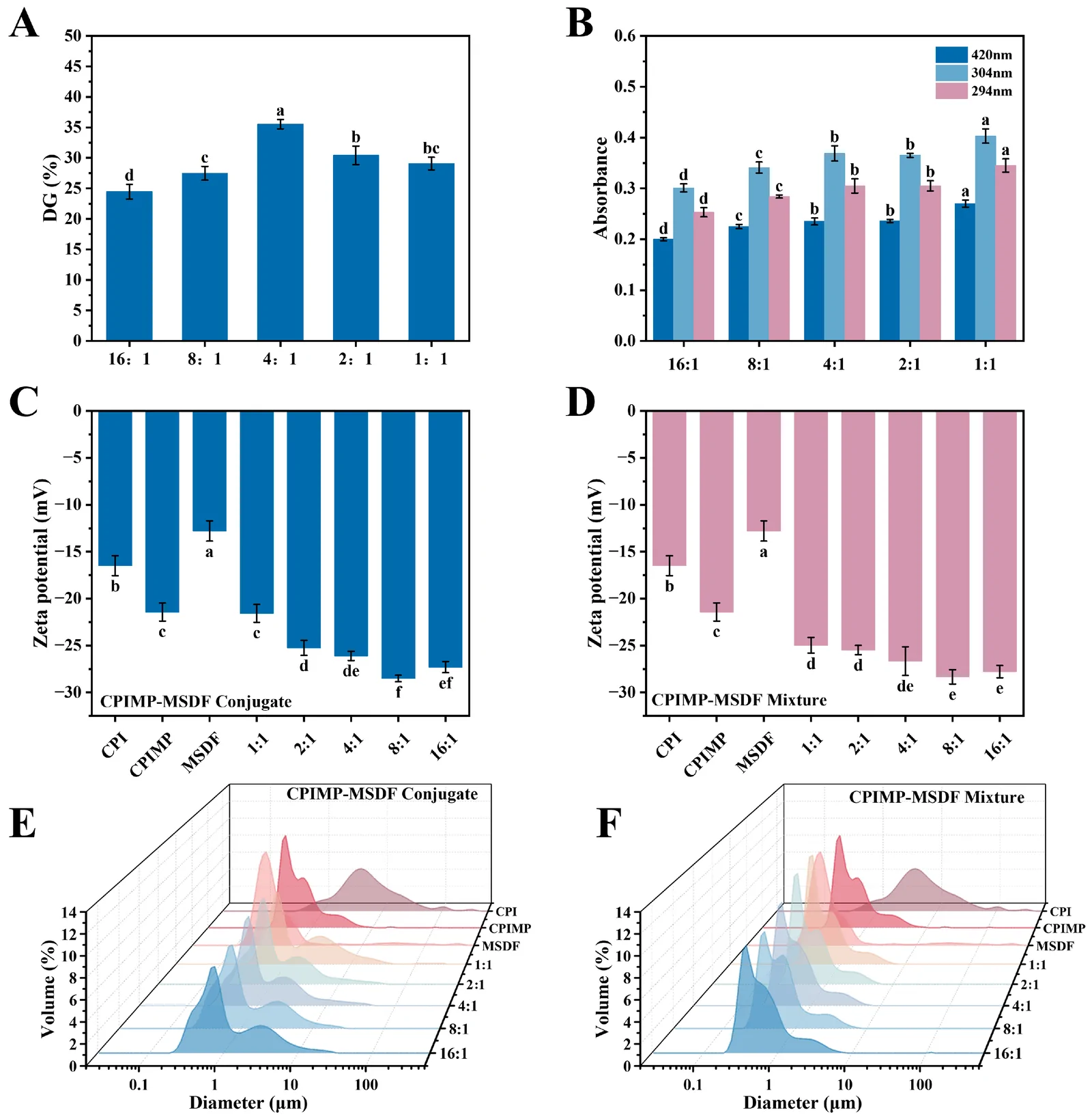
Degree of grafting (DG) (**A**) and browning intensity (**B**) of CPIMP–MSDF conjugates prepared at different mass ratios; zeta potential (**C**,**D**) and particle size (**E**,**F**) of CPIMP–MSDF mixtures and CPIMP–MSDF conjugates prepared at different mass ratios. Different letters represent significant differences between groups (*p* < 0.05).

The Maillard reaction between CPIMP and MSDF mainly relies on the condensation reaction between ε-amino groups of the protein and carbonyl groups at the polysaccharide reducing ends. As presented in Figure 1A, the DG of CPIMP–MSDF conjugates rose initially and then fell as the polysaccharide ratio increased, reaching its peak (35.5%) at a CPIMP-to-MSDF mass ratio of 4:1. CPIMP was prepared via the high-pressure homogenization and ultrasound pretreatment of CPI gels to facilitate the conformational unfolding of CPI and improve the DG. Furthermore, as the proportion of MSDF increases (from 16:1 to 4:1), more carbonyl groups react with CPIMPs, resulting in a higher DG. The DG of ozone-pretreated CPI-arabinose conjugates exhibited the same trend, first increasing and then decreasing with increasing arabinose proportion [34]. However, an excessive concentration of MSDF may enhance its own steric hindrance effect, impairing the mobility and collision between protein and polysaccharide molecules, and hindering the contact between free amino groups and carbonyl groups [35]. As reported by Liu et al. [36], the grafting of polysaccharides onto proteins may be hampered by the elevated solution viscosity associated with increased MSDF concentration.

The glycation of proteins can be divided into three stages: early, intermediate, and late. In the late stage, brown, water-soluble heterogeneous polymers called melanoidins are formed, which are the main substances responsible for the browning intensity. The absorbance at 294 nm (A_294_) is used to characterize the glycosylation intermediates (such as hydroxymethylfurfural, reductones, and aldehydes) generated at the initial reaction phase. The absorbance at 304 nm (A_304_) represents the accumulation of Amadori products in the intermediate stage. The absorbance at 420 nm (A_420_) indicates the formation intensity of melanoidins, the browning products in the late stage. Together, these three parameters comprehensively reflect the progression of Maillard glycation. As illustrated in Figure 1B, the A_294_, A_304_ and A_420_ of the CPIMP–MSDF conjugates all increased with increasing MSDF proportion. The increase in absorbance may be attributed to excess polysaccharide, thereby accelerating the accumulation of intermediates and promoting the formation of brown substances [37]. The higher A_294_ than A_420_ indicates that the reaction system is dominated by early-stage Maillard products. Moreover, the ultrasonic pretreatment inhibited the polymerization of intermediates to some extent, thereby reducing the browning intensity of the CPIMP–MSDF conjugates [38]. This study focused on the overall performance of the combined homogenization-ultrasonication pretreatment for microgel fabrication. We note that the present experimental design cannot fully separate the individual contribution of homogenization or ultrasonication alone. Future investigations will adopt single-factor experiments with individual treatment controls to clarify their separate roles in protein structural modification.

#### 3.1.2. Zeta-Potential and Particle Size

Figure 1C,D present the zeta-potential results for CPI, CPIMP, the CPIMP–MSDF mixtures, and the CPIMP–MSDF conjugates. The 4:1 CPIMP–MSDF conjugate with the highest grafting degree showed a relatively high absolute zeta potential of 26.68 mV. The covalent conjugation between CPIMPs and MSDF consumed more amino groups, thereby increasing the amount of negative surface charges carried by protein molecules [39]. This higher absolute zeta potential endows the conjugate with intensified electrostatic repulsion, which helps inhibit droplet aggregation and maintain system stability. In contrast, the absolute zeta potential of CPIMPs exceeded that of the native CPI, which was due to the formation of smaller protein aggregates and conformational changes resulting from high-pressure homogenization and ultrasonication, thereby exposing more charged groups on the protein surface. Meanwhile, the CPIMP–MSDF mixture exhibited a higher absolute zeta potential at the same ratio than the CPIMP–MSDF conjugate, possibly due to stronger electrostatic repulsion between molecules in the physically mixed system [40].

The particle size distributions of the CPIMP–MSDF conjugates and mixtures at various concentrations changed compared with that of CPIMPs (Figure 1E,F). This can be explained by the steric hindrance effect arising from the addition or grafting of MSDF, which effectively reduces protein–protein interactions and prevents particle aggregation [41]. Compared with the CPIMP–MSDF mixtures, the CPIMP–MSDF conjugates showed a marked change in particle size distribution. This may be partially attributed to disulfide bond rearrangement induced by high-temperature incubation. Meanwhile, covalent linkages formed during the Maillard reaction may connect different molecules, which could help suppress disordered aggregation driven by hydrophobic interactions or hydrogen bonding and favor the formation of stable colloidal dispersions [42].

#### 3.1.3. FTIR Spectroscopy and Protein Secondary Structure Analysis

**Figure 2:**
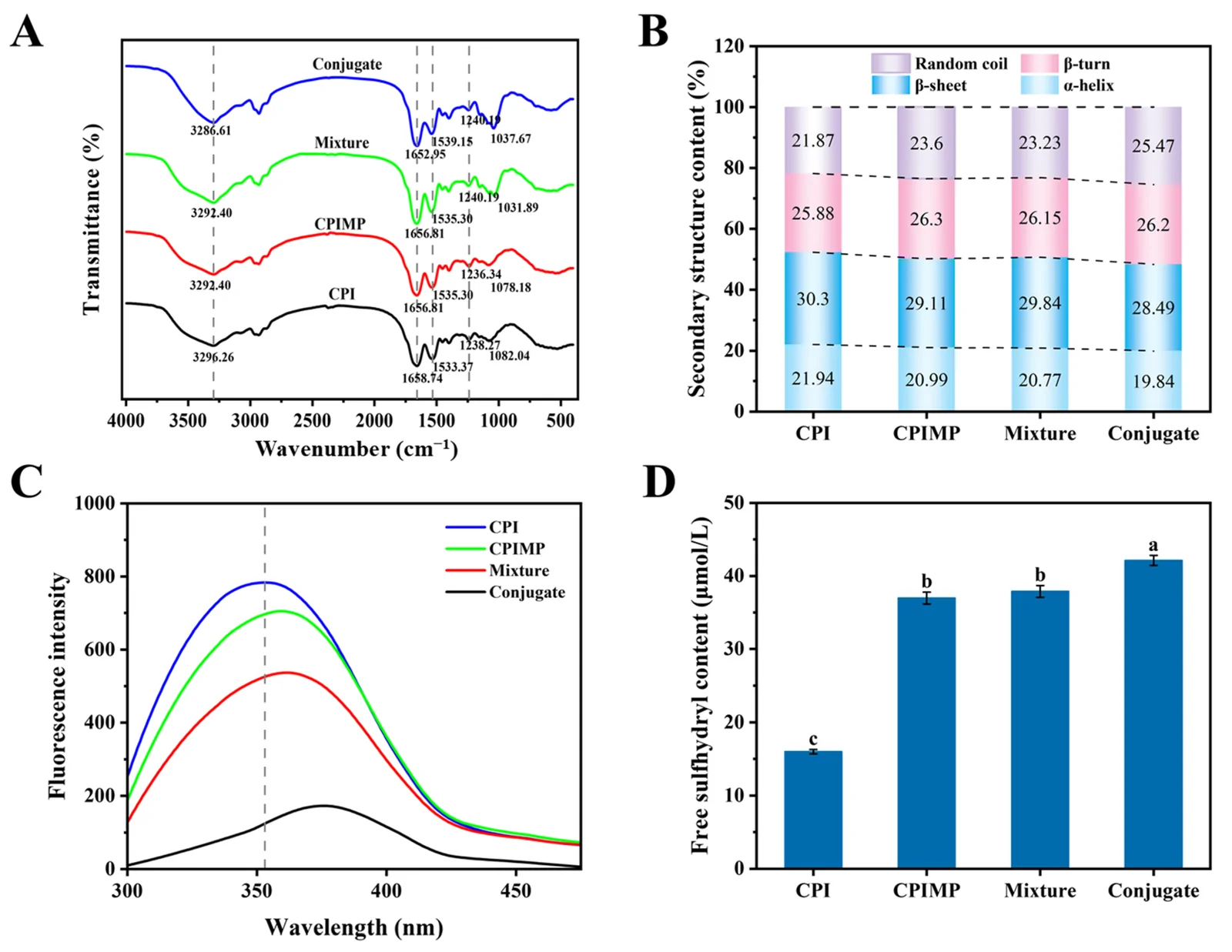
FTIR spectra (**A**), secondary structure (**B**), fluorescence spectra (**C**), and free sulfhydryl content (**D**) of CPI, CPIMP, CPIMP–MSDF mixture, and CPIMP–MSDF conjugate. Different letters represent significant differences between groups (*p* < 0.05).

FTIR is commonly employed to identify the functional group alterations resulting from protein–polysaccharide interactions, which can reveal the structural changes. As illustrated in Figure 2A, a broad absorption peak was observed at 3750–3000 cm^−1^ in the spectrum of CPI, which was associated with O–H and N–H stretching vibrations, confirming the involvement of hydrogen bonds. Additionally, two weak signals at 2928.46 and 2858.46 cm^−1^ corresponded to the stretching of saturated C–H bonds [43]. The main absorption peaks of CPI at 1658.74 cm^−1^, 1533.37 cm^−1^, and 1238.27 cm^−1^ corresponded to the amide I band (C=O stretching), amide II band (C–N stretching vibration and N–H bending vibration), and amide III band (C–N stretching and N–H bending), respectively [30]. Relative to CPI, the C–O stretching band of CPIMPs moved from 3296.26 cm^−1^ to 3286.61 cm^−1^, and the amide I and III bands exhibited a red shift, indicating increased hydrogen bonding at the molecular level. This could be attributed to the alteration of CPI’s internal structure after fabrication into microgels via high-pressure homogenization shearing and ultrasound pretreatment. Compared with CPIMP, both the CPIMP–MSDF mixture and the CPIMP–MSDF conjugate exhibited shifts in the O-H stretching vibration band, as well as the amide I and II bands, indicating an interaction between CPIMP and MSDF. Compared with the CPIMP–MSDF mixture, the peak shift in the CPIMP–MSDF conjugate was more pronounced and shifted from 3292.04 cm^−1^ to 3682.61 cm^−1^, possibly due to the glycation effect [44]. The consumption of –NH_2_ groups and the generation of Amadori compounds (C=O), Schiff base (C=N) and Pyrazines (C–N) products during glycation led to the peak intensity changes in the conjugate, which primarily affected the C–O and C–N stretching and N–H bending vibrations [45]. Additionally, a notable red shift accompanied the enhanced band of the conjugate at 1100–1020 cm^−1^, which was linked to the C–O–C and C–O–H stretching vibrations. Similarly, a significantly enhanced peak at 1050–1150 cm^−1^ was reported for the soybean protein isolate-chitosan oligosaccharide complex, which suggests binding interactions between the protein and the polysaccharide molecule [10].

The amide I band in the region of 1600–1700 cm^−1^ was fitted using PeakFit software to analyze the secondary structure of the protein, including α-helix (1610–1640 cm^−1^), β-sheet (1640–1650 cm^−1^), β-turn (1650–1660 cm^−1^), and random coil (1660–1700 cm^−1^) [46]. In Figure 2B, the secondary structure of CPI consisted of 21.94% α-helix, 30.3% β-sheet, 25.88% β-turn, and 21.87% random coil. Compared to CPI, the α-helix and β-sheet contents of CPIMP slightly decreased but the β-turn and random coil contents slightly increased. The CPIMP particles prepared by high-pressure homogenization and ultrasound have a looser spatial structure. For the glycated CPIMP–MSDF conjugate, the α-helix and β-sheet contents showed a marked decrease, while the random coil content exhibited a significant increase from 21.87% to 25.47%. This indicates that glycosylation may disrupt the original hydrogen bonding network and hydrophobic interactions, thereby promoting the unfolding of the polypeptide chain. Consistent with previous reports, CPI covalently bound to citrus pectin showed a decrease in α-helix and an increase in random coil, indicating that glycation resulted in a looser protein structure [36]. The functionality is tightly linked to the conformational structure. The unfolded CPIMP was more prone to grafting with MSDF via the Maillard reaction, and the resulting glycated CPIMP–MSDF conjugate possessed a more flexible spatial conformation. Consequently, the interfacial adsorption of the conjugates is facilitated, which may improve their emulsifying performance [47].

#### 3.1.4. Intrinsic Fluorescence Spectroscopy

The intrinsic fluorescence spectra with tryptophan as the emitting group can reflect the differences in the tryptophan microenvironment between native CPI and the samples after CPI microgelation and MSDF modification [48]. Figure 2C shows that the fluorescence emission maximum of CPI appeared at 353.07 nm, while that of CPIMP red-shifted to 359.07 nm with a markedly reduced fluorescence intensity relative to CPI. This indicates that the protein conformation was altered upon gelation, leading to the shifting of some tryptophan residues toward a more polar microenvironment. Concurrently, the fluorophore was altered, resulting in quenching of the chromophore [49]. The CPIMP–MSDF mixture exhibited lower fluorescence intensity than CPIMP. Furthermore, the CPIMP–MSDF conjugate decreased even more. This decrease is likely due to the quenching of protein fluorescence by the shielding effect of MSDF. The fluorescence intensity of the glycinin-soybean soluble polysaccharide conjugate was higher than that of the glycinin-polysaccharide mixture, and higher than that of pure glycinin [50]. The CPIMP–MSDF conjugate exhibited a more pronounced red shift in its fluorescence spectrum, indicating that the Maillard reaction may induce a looser protein structure along with the relocation of tryptophan residues to a more polar and hydrophilic microenvironment. This also suggests a certain degree of unwinding of the protein tertiary structure.

#### 3.1.5. Free Sulfhydryl Content

Sulfhydryl groups are key functional groups in proteins, which stabilize the tertiary structure through the formation of disulfide bonds. Figure 2D shows that the free sulfhydryl content of the CPIMP increased to 36.97 μmol/L compared with that of CPI (15.97 μmol/L). The preparation of CPIMP from CPI gel via high-pressure homogenization shearing and ultrasonication induces significant conformational unfolding of the protein, exposing cysteine residues from the hydrophobic core and cleaving some disulfide bonds, thus greatly increasing the free sulfhydryl content. More importantly, the CPIMP–MSDF conjugate possessed the greatest free sulfhydryl content. The structural unfolding of CPIMP exposes more internal sulfhydryl groups within the protein microgel, which may be associated with the heat treatment and Maillard reaction-related structural rearrangement [51]. A study showed that when soy protein isolate was covalently conjugated with goji berry polysaccharide, the free sulfhydryl content increased with increasing polysaccharide concentration [52].

### 3.2. Functional Characterizations of CPIMP–MSDF Conjugate

#### 3.2.1. DPPH• Radical Scavenging Activity

**Figure 3:**
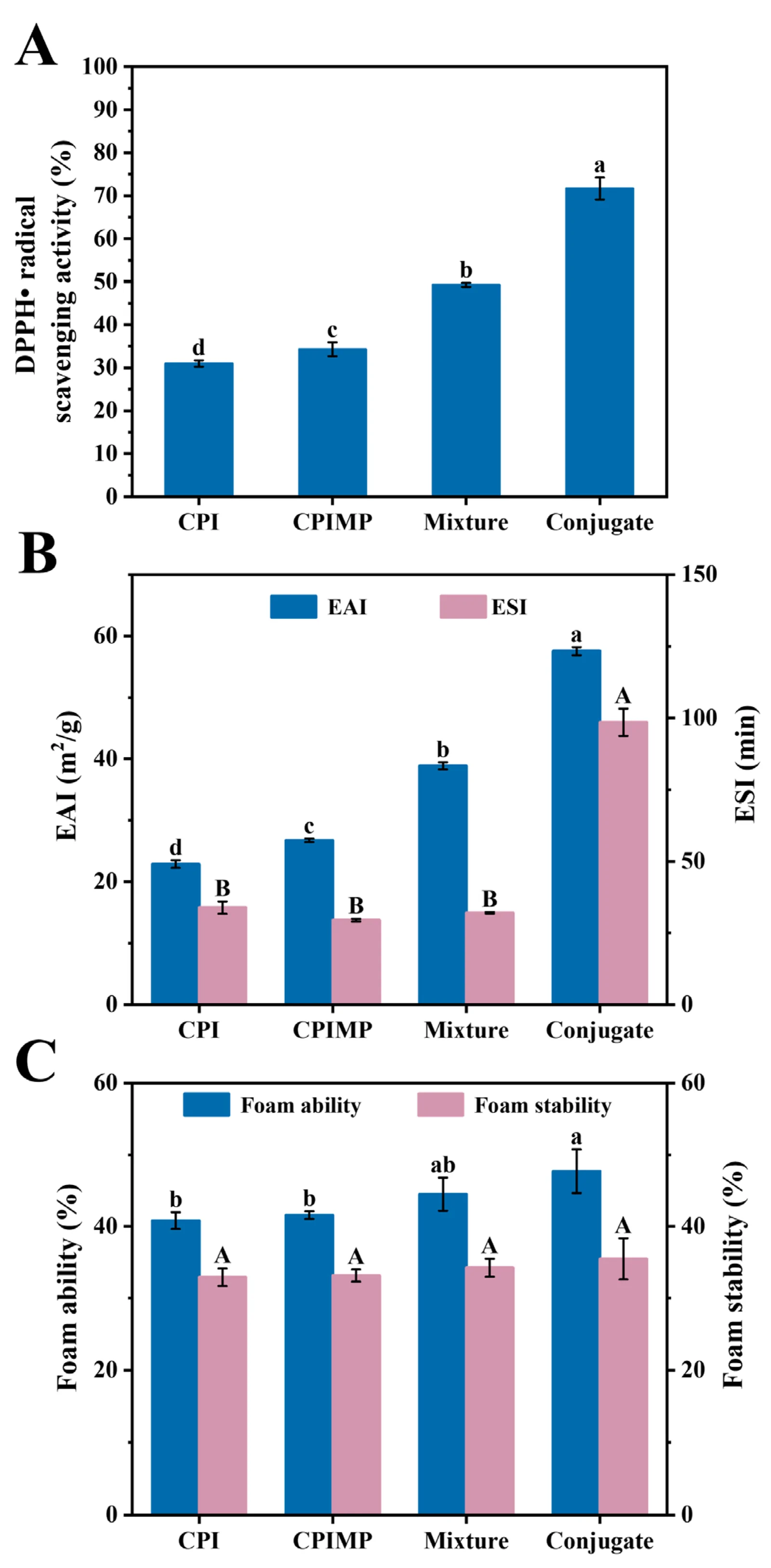
DPPH• radical scavenging activity (**A**), emulsifying activity index (EAI) and emulsifying stability index (ESI) (**B**), foaming capacity and foam stability (**C**) of CPI, CPIMP, CPIMP–MSDF mixture, and CPIMP–MSDF conjugate. Different letters represent significant differences between groups (*p* < 0.05).

DPPH• can be reduced by antioxidants through electron or hydrogen transfer, and thus it is widely used for evaluating the antioxidant capacity of different samples. As illustrated in Figure 3A, The DPPH• radical scavenging activities of CPI, CPIMP, CPIMP–MSDF mixture, and CPIMP–MSDF conjugate were in the order of CPI < CPIMP < Mixture < Conjugate (*p* < 0.05). The CPIMP–MSDF conjugate achieved a significantly higher antioxidant activity (71.70%) than the other samples, suggesting that Maillard-mediated covalent conjugation may markedly enhance its free radical scavenging capacity. Similar findings were demonstrated that the covalent conjugate of flaxseed gum and whey protein formed via the Maillard reaction exhibited markedly enhanced antioxidant activity [53]. Covalent grafting of proteins with polysaccharides significantly promotes the unfolding of secondary and tertiary structures, exposing more reactive residues and thereby enhancing the hydrogen-donating capacity of proteins. Another study reported that the increased DPPH• scavenging activity of β-lactoglobulin upon heating with reducing glucose was attributed to the caramelization of glucose and the formation of melanoidins, and melanoidins are known to exhibit antioxidant activity [31]. Moreover, the antioxidant activity of CPIMP is higher than that of native CPI, which may be ascribed to the structural changes in proteins during gel shearing. The higher antioxidant activity of the CPIMP–MSDF mixture relative to CPIMP likely resulted from the cooperative action of CPIMP and MSDF [54]. On one hand, previous studies have illustrated that mung-bean-hull-derived MSDF naturally contains abundant reducing sugars and polyphenols endowed with antioxidant properties [24,25]. On the other hand, MSDF might interact with CPIMP through hydrogen bonding and hydrophobic forces, which further improve antioxidant capacity. Considering that MSDF comprises carbohydrates, reducing sugars, residual protein and intrinsic polyphenols reported in the literature, these moieties may collectively contribute to reducing and antioxidant behaviors in our system. Given that only CPIMP–MSDF conjugates and physical mixtures were compared in the current experimental setup, it remains challenging to fully deconvolute the exact origin of the observed improvement in antioxidant activity. Further work will isolate and quantify the respective contributions of reducing sugar and phenolic fractions.

#### 3.2.2. Emulsifying Properties

The ability of an emulsifier to form emulsions is expressed as the emulsifying activity index (EAI), whereas the resistance of the emulsions to coalescence and flocculation is reflected by the emulsion stability index (ESI). In Figure 3B, the EAI of CPIMP was higher than that of CPI, which can be attributed to the improved water solubility and amphiphilicity resulting from the preparation of CPIMP microgels via high-pressure homogenization and ultrasonication, as well as the excellent interfacial activity of CPIMP as a soft colloidal particle. The CPIMP–MSDF mixture showed improved emulsifying activity, which was due to the enhanced steric hindrance and electrostatic repulsion from MSDF. Moreover, the CPIMP–MSDF conjugate showed the highest EAI (57.54 m^2^/g) and ESI (98.50 min), which were 2.52- and 2.91- fold higher than those of CPI, respectively. This enhancement in emulsifying performance can be attributed to three main factors. (1) The hydrophilic group content in the conjugate was elevated by the glycosylation reaction, which significantly improved protein solubility and accelerated its migration and adsorption at the interface [55]. (2) The grafted polysaccharide chains may form a thick interfacial layer, which helps prevent droplet aggregation and flocculation [34]. (3) The glycosylated conjugate exhibited stronger electrostatic repulsion, further enhancing droplet dispersibility and stability [56].

#### 3.2.3. Foaming Properties

Foaming properties and emulsifying properties are intrinsically linked, as both are governed by protein solubility and amphiphilicity. It has been extensively reported that the covalent grafting of saccharides onto the surface of proteins substantially enhances the foamability of these proteins [33]. The CPIMP–MSDF conjugate exhibited the highest foamability among all samples, reaching 121.49% (Figure 3C). The structural changes induced by protein–polysaccharide conjugation increase the intermolecular gaps and molecular mobility of the protein, which accounts for the enhanced foaming ability. Covalent grafting of polysaccharides increases the solubility and dispersibility of protein molecules, which is crucial for foam formation and stabilization. For example, the ovalbumin-carboxymethyl cellulose conjugate exhibited significantly improved foaming capacity compared with native ovalbumin, owing to the enhanced solubility and flexibility of ovalbumin after conjugation [55].

### 3.3. Emulsion Properties Stabilized by CPIMP–MSDF Conjugate

#### 3.3.1. Microstructure Observation (CLSM)

**Figure 4:**
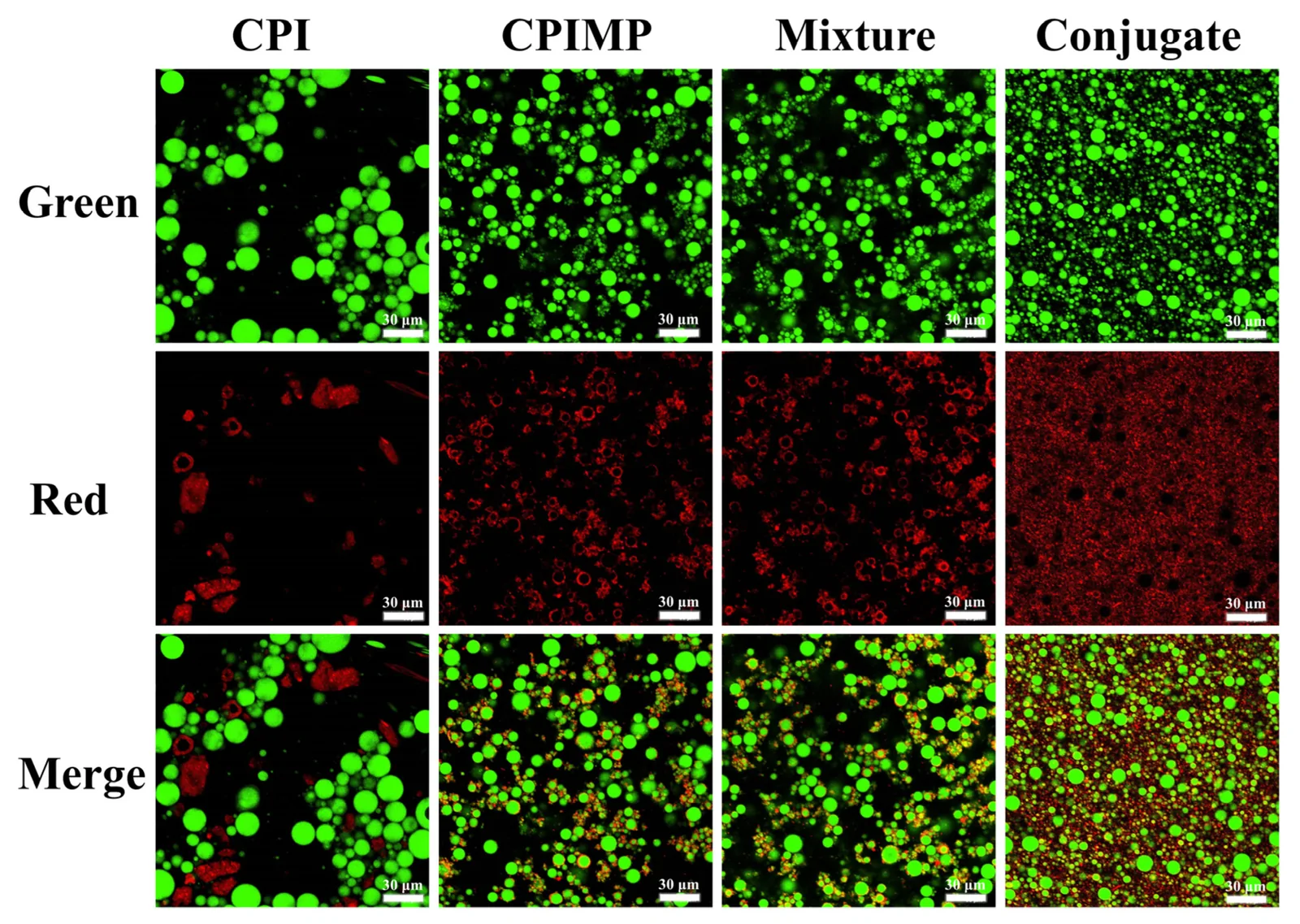
Microstructures (CLSM) of the freshly prepared emulsions stabilized by CPI, CPIMP, CPIMP–MSDF mixture, and CPIMP–MSDF conjugate.

The CLSM microstructures of emulsions stabilized by CPI, CPIMP, the CPIMP–MSDF mixture, and the CPIMP–MSDF conjugate are depicted in Figure 4. The green fluorescence image represents oil droplets stained with Nile Red, and the red fluorescence area represents protein images stained with Nile Blue. The red fluorescence of emulsions stabilized by CPIMP, the CPIMP–MSDF mixture and the CPIMP–MSDF conjugate are all distributed around the green fluorescence, indicating effective adsorption of the covalently modified proteins onto the oil droplet surface and the formation of an interfacial membrane. The CLSM observations are consistent with previous studies, further confirming that the CPIMP–MSDF conjugate possesses relatively excellent emulsifying properties. The CPIMP–MSDF conjugates exist as colloidal particles in the continuous phase instead of soluble macromolecular chains. Glycosylation modification of endows proteins with enhanced amphipathic properties, enabling the formation of a dense interfacial film constructed from protein–polysaccharide composites. Meanwhile, this interfacial membrane formed by the conjugate exhibits stronger steric hindrance and electrostatic repulsion, which effectively prevents droplet coalescence [57].

#### 3.3.2. Particle Size and Zeta-Potential

**Figure 5:**
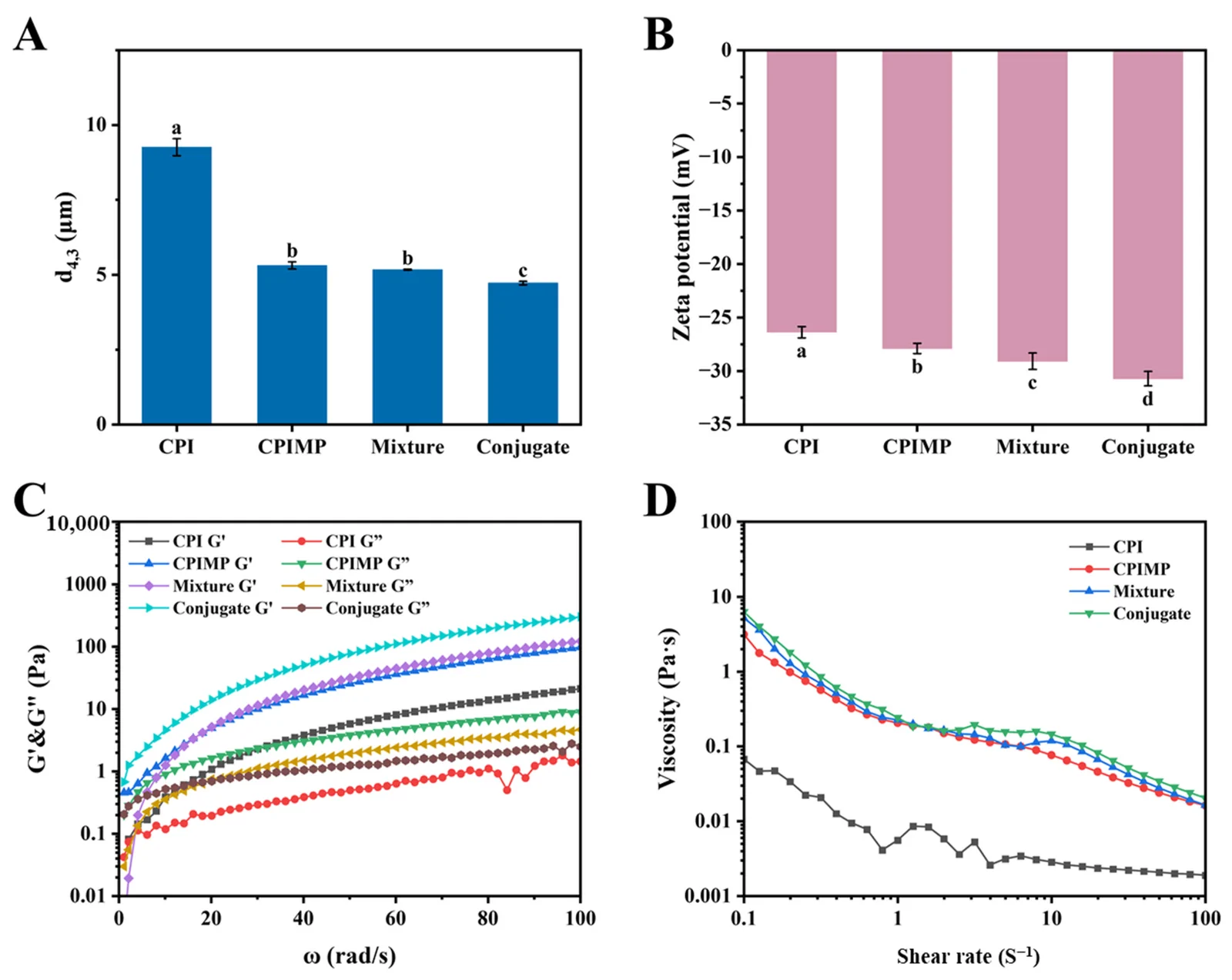
The average droplet size d_4,3_ (**A**), zeta potential (**B**), frequency scan curves (**C**) and apparent viscosity (**D**) of emulsions stabilized by CPI, CPIMP, CPIMP–MSDF mixture, and CPIMP–MSDF conjugate. Different letters represent significant differences between groups (*p* < 0.05).

As presented in Figure 5A,B, the mean particle diameter (d_4,3_) and zeta potential measurements confirmed the microstructural differences among the emulsions. The freshly prepared CPI-stabilized emulsion exhibited a d_4,3_ of 9.26 μm. However, the CPIMP-stabilized emulsion showed a significantly reduced d_4,3_ of 5.31 μm, owing to the unfolded protein structure of CPIMP prepared via combined high-pressure homogenization and ultrasonication pretreatment, which effectively inhibited droplet coalescence and flocculation. In contrast, the CPIMP–MSDF conjugate-stabilized emulsion exhibited the smallest d_4,3_ of 4.72 μm. This can be attributed to the enhanced interfacial adsorption of glycosylated proteins, which were able to form a compact interfacial layer and facilitated uniform droplet dispersion [58]. The emulsions showed a gradual decrease in zeta potential from −24.86 mV (CPI) to −27.29 mV (CPIMP). After glycation, the CPIMP–MSDF conjugate exhibited the most negative value (−30.71 mV), which can be attributed to the increase in negatively charged surface moieties, the attenuation of ion shielding effects, and the exposure of internal charged residues [36]. The particle size data confirmed that CPIMP–MSDF existed as colloidal particles dispersed in water rather than soluble macromolecules. Combined with microscopic observations, these colloidal particles adsorbed on oil droplet surfaces and formed a particulate interfacial barrier, which demonstrated the characteristic particulate stabilization mechanism of Pickering emulsions.

#### 3.3.3. Rheological Properties

The storage modulus (G′) and loss modulus (G″) of emulsions stabilized by different emulsifiers were measured (Figure 5C). As the angular frequency increased, both G′ and G″ exhibited a gradual rise, suggesting distinct frequency-dependent behavior. Moreover, G′ exceeded G″ over the entire frequency range, indicating a predominantly elastic, gel-like characteristic. The CPIMP–MSDF conjugate-stabilized emulsion showed significantly higher G′ and G″ than the other samples, indicating a more robust viscoelastic network of the whole emulsion system, which may be partially attributed to the particulate interfacial layer assembled at the oil-water interface. In Figure 5D, the viscosity of all emulsions decreased steadily with increasing shear rate, exhibiting typical shear-thinning behavior. Such flow behavior generally arises from the breakdown of internal networks, flocculation of droplets, and disentanglement of biopolymer chains under shear [59]. Notably, the emulsions stabilized by the CPIMP, the CPIMP–MSDF mixture and the CPIMP–MSDF conjugate all exhibited relatively high apparent viscosity. The formation of protein microgels largely accounted for the increased overall viscosity of the system. Additionally, the enhanced steric hindrance together with electrostatic repulsion provided by the polysaccharide chains effectively maintained a fine and uniform droplet distribution.

#### 3.3.4. Storage Stability of the Emulsions

**Figure 6:**
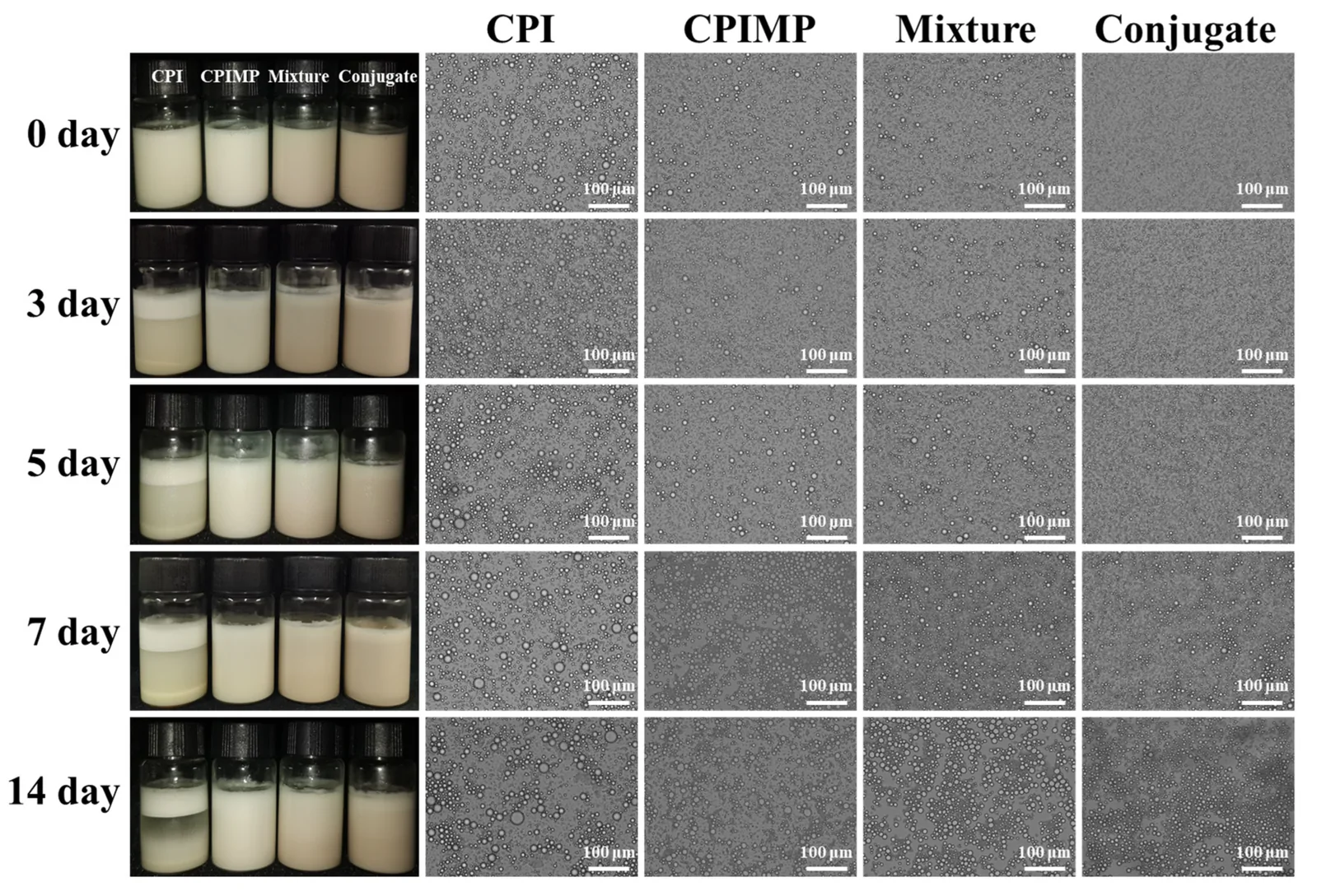
Appearance and optical microscopic image of the emulsions after storage for 0, 3, 5, 7 and 14 days.

The appearance and optical microscopy images of different emulsions after storage for 0, 3, 5, 7, and 14 days are presented in Figure 6. Freshly prepared emulsions all exhibited relatively uniform droplet distribution. By contrast, after 3 days of storage, the CPI emulsion showed signs of phase separation accompanied by partial droplet aggregation. By day 14, clear oil-water phase separation was observed in the CPI emulsion. In contrast, emulsions prepared by CPIMP, the CPIMP–MSDF mixture, and the CPIMP–MSDF conjugate exhibited no phase separation during storage, indicating significantly improved emulsion stability.

**Figure 7:**
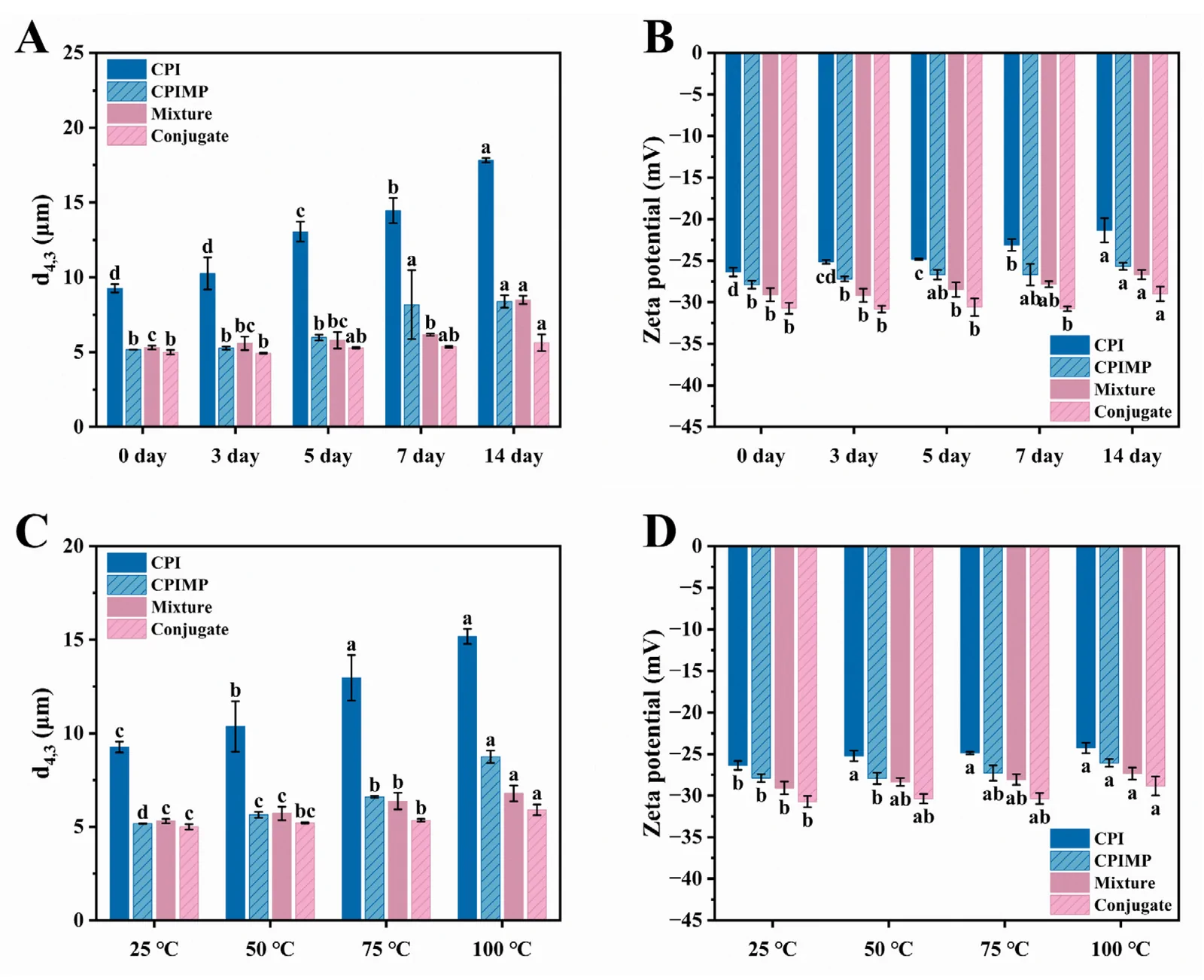
Effects of storage time (0, 3, 5, 7, and 14 days) on the average droplet size d_4,3_ (**A**) and zeta potential (**B**) of emulsions. Effects of heat treatment at different temperatures (25, 50, 75, and 100 °C) on the average droplet size d_4,3_ (**C**) and zeta potential (**D**) of emulsions. Different lowercase letters indicate significant differences at *p* < 0.05.

The two-way ANOVA revealed that both formulation and storage time exerted significant main effects on droplet size and zeta potential, and a significant interaction was also observed between these two factors (*p* < 0.05). Figure 7A and Figure 7B respectively show the alterations in d_4,3_ and zeta potential of different emulsions over storage time. The CPIMP–MSDF conjugate-based emulsion displayed outstanding stability throughout the 14-day storage period, with the smallest initial droplet size (4.72 μm) that increased only slightly to 5.64 μm by day 14. Meanwhile, the absolute zeta potential of the emulsion system dropped from 30.71 mV to 28.99 mV over the 14 days, consistently maintaining the highest absolute value among all samples. This indicates that the CPIMP–MSDF conjugate formed a dense and stable protein–polysaccharide composite interfacial layer on the oil droplet surface, effectively inhibiting droplet coalescence. In contrast, the CPI-stabilized emulsion showed a continuous and pronounced increase in droplet size, reaching 17.83 μm by day 14, while its absolute zeta potential dropped to 21.34 mV on the same day. Compared with CPI, the emulsions stabilized by CPIMP and the CPIMP–MSDF mixture exhibited relatively minor changes over the storage period. Therefore, the structural rearrangement and enhanced steric hindrance induced by microgel formation and polysaccharide conjugation endowed the CPIMP–MSDF conjugate-stabilized emulsion with outstanding stability.

#### 3.3.5. Effect of Heating on Emulsions Stability

The two-way ANOVA revealed that both formulation and heating temperature exerted significant main effects on droplet size and zeta potential, and a significant interaction was observed between these two factors (*p* < 0.05). Thermal processing, including sterilization and boiling, is a critical step in the food industry for ensuring product quality and safety. Consequently, the thermal stability of the freshly prepared emulsions were evaluated after treatment at different temperatures (25, 50, 75, and 100 °C) for 3 h in this study, and the resulting droplet size and zeta potential data are presented in Figure 7C,D. The CPI-stabilized emulsion showed an increase from 9.26 to 15.18 μm, while the CPIMP–MSDF conjugate-stabilized emulsion exhibited only a 1.19 μm increase. Thermal treatment enhances Brownian motion and increases collision frequency among oil droplets, thereby accelerating their aggregation or coalescence [60]. Notably, heat treatment not only disrupts protein structure but also reduces the absolute zeta potential, thereby impairing interfacial adsorption and emulsion-stabilizing ability, which may ultimately lead to phase separation. The covalent conjugation of MSDF with CPIMP enhances interfacial adsorption, forming a thickened interfacial film that inhibits droplet aggregation and improves emulsion thermal stability.

## 4. Conclusions

In summary, this study evaluated the CPIMP–MSDF conjugate formed via a wet-heating Maillard reaction. This conjugate exhibited significantly improved structural, functional, and emulsifying properties. CPIMPs were fabricated by gelation followed by combined homogenization and ultrasonication pretreatment, which effectively modified the molecular conformation of CPI and substantially improved the DG of the resulting CPIMP–MSDF conjugates. FTIR, intrinsic fluorescence spectroscopy, and free sulfhydryl content collectively suggest the occurrence of Maillard-mediated covalent grafting between CPIMP and MSDF at the molecular level. These structural changes modulated the surface properties and interfacial adsorption behavior of the protein, leading to significantly enhanced antioxidant activity, emulsifying properties, and foaming properties. Compared with CPI, CPIMP, and the CPIMP–MSDF mixture, the CPIMP–MSDF conjugate-stabilized Pickering emulsion exhibited the smallest droplet size and enhanced environmental stability. Collectively, glycosylation modification of CPIMP effectively improves its interfacial activity and emulsifying performance, overcoming the inherent limitations of native plant proteins, such as structural instability and weak interfacial adsorption capacity. The CPIMP–MSDF conjugate prepared in this study represents a promising green plant-based emulsifier with broad application potential in functional emulsions and bioactive delivery systems.

## Data Availability

The original data generated in this study are contained within this article. Any further questions may be addressed to the corresponding authors.

## Abbreviations

The following abbreviations are used in this manuscript:

CPI	Chickpea Protein Isolate
CPIMP	Chickpea Protein Isolate Microgel Particles
MSDF	Mung Bean Hull Soluble Dietary Fiber
EAI	Emulsifying Activity Index
ESI	Emulsion Stability Index
